# Maternal Smoking During Pregnancy and Growth in Infancy: a Covariance Structure Analysis

**DOI:** 10.2188/jea.JE20140040

**Published:** 2015-01-05

**Authors:** Wei Zheng, Kohta Suzuki, Ryoji Shinohara, Miri Sato, Hiroshi Yokomichi, Zentaro Yamagata

**Affiliations:** 1 Department of Health Sciences, Interdisciplinary Graduate School of Medicine and Engineering, University of Yamanashi, Chuo, Yamanashi, Japan; 1 山梨大学大学院医学工学総合研究部 社会医学講座; 2 Center for Birth Cohorts Studies, Interdisciplinary Graduate School of Medicine and Engineering, University of Yamanashi, Chuo, Yamanashi, Japan; 2 山梨大学大学院医学工学総合研究部附属出生コホート研究センター

**Keywords:** child development, maternal exposure, smoking

## Abstract

**Background:**

Smoking during pregnancy is related to fetal constraint and accelerated postnatal growth. However, the pathways between these factors have not been clarified. Pathway analyses that link these factors can help us better understand the mechanisms involved in this association. Therefore, this study aimed to examine pathways between maternal smoking during pregnancy and growth in infancy.

**Methods:**

Participants were singletons born between 1993 and 2006 in rural Japan. The outcome was the change in weight z-score between birth and 3 years of age. Pathways from maternal smoking and other maternal factors (such as maternal body mass index and work status) to growth in infancy via birth factors (such as birth weight and gestational age) and breastfeeding were examined using structural equation modeling.

**Results:**

Complete data were available for 1524 children (775 boys and 749 girls). The model fit appeared adequate. Lower birth weight and non-exclusive breastfeeding mediated the association between maternal smoking during pregnancy and rapid growth in infancy. Maternal smoking was also directly linked to rapid growth in infancy (standardized direct effects 0.06, *P* = 0.002). Taking all pathways into account, the standardized total effect of maternal smoking on growth in infancy was 0.11.

**Conclusions:**

Maternal smoking during pregnancy may both indirectly, through birth weight and breastfeeding status, and directly influence growth during infancy; however, there may be other pathways that have not yet been identified.

## Abstract (ja)

**背景：:**

妊娠中の喫煙は胎児の発育制限と出生後の急激な発育に関連していたが、これらの因子間の経路はまだ明らかにされていない。これらの因子における経路の解析は、妊娠中の喫煙が胎内発育、そして乳幼児期の発育に及ぼす影響のメカニズムを解明するために重要である。本研究では、妊娠中の喫煙と出生児における乳児期の成長との間の経路を調べることを目的とした。

**方法：:**

参加者は、1993年から2006年の間に日本の一地域で生まれた単胎児である。出生体重と3歳の体重における標準偏差スコアの変化を主要アウトカムとした。母親の喫煙、他の母親に関する因子（母親のBody Mass Indexと就労状況）、出生時の因子（出生体重と在胎週数）と母乳育児のそれぞれが、どのように幼児期の成長と関連しているかを、構造方程式モデリングを用いて検討した。

**結果：:**

1524人の参加者（男児775人、女児749人）から欠損値のないデータが得られ、解析モデルは適切と考えられた。出生体重が軽いこと、完全母乳育児でないことは、妊娠中の喫煙と幼児期の急激な発育の関連における媒介因子であった。一方で、母親の喫煙と、乳児期の成長との直接的な関連も観察された（standardized direct effects: 0.06, p = 0.002）。すべての経路を考慮した後の、幼児期の成長に与える妊娠中の喫煙の標準化した寄与率は0.11であった。

**結論：:**

妊娠中の喫煙は、出生体重と母乳育児を経由して、間接的に幼児期の成長に影響する経路と、直接的に幼児期の成長に影響する経路の双方が存在する可能性が示された。さらに、他のまだ同定されていない経路が存在するかもしれない。

## INTRODUCTION

Appropriate growth during infancy, particularly the rate of growth, is critical to health in later life. Systematic reviews have consistently identified an association between rapid weight gain in infancy and later obesity.^1^^–^^3^ Further, a study carried out in Barcelona by Ibáñez et al demonstrated that rapid weight gain in infancy is associated with central adiposity and insulin resistance.^4^ Another study conducted in the Netherlands indicated that rapid weight gain during very early infancy is associated with several determinants of cardiovascular disease and type 2 diabetes in early adulthood.^5^ Given the results of these studies, it is important to identify the factors that might be associated with rapid growth during infancy. Therefore, numerous studies have explored the factors that may influence growth during this period.

Relationships have been reported between growth in infancy and maternal factors such as maternal smoking during pregnancy,^6^ in addition to birth and early-life factors such as birth weight,^7^ gestational age, and feeding methods.^8^ However, these factors may also be interrelated. In addition, birth factors might mediate the effect of parental factors. These maternal and early-life factors work together to influence growth in infancy. To better understand the effect of each factor and the potential interactions among factors, we attempted to identify the pathways between maternal factors, birth and early-life factors, and growth in infancy using pathway analysis.

Among these factors, maternal smoking during pregnancy has been reported as a major cause of fetal undernutrition and has been shown to be related to later obesity.^9^ However, information on mechanisms through which maternal smoking during pregnancy affects fetal and postnatal growth is lacking; further, other factors that may influence these relationships have not been thoroughly studied. Therefore, in this study, we examined the pathways between maternal smoking during pregnancy and offspring growth in infancy after controlling for other factors that could also influence growth during infancy.

## METHODS

### Study sample

The study participants comprised children born between April 1, 1993, and March 31, 2006, in the Enzan area of Koshu City, Yamanashi Prefecture, Japan. The participants were from Project Koshu, which is a dynamic Japanese community-based prospective cohort study. Project Koshu is an ongoing study that started in 1988, in which all expectant mothers who responded to a survey during the obligatory visit at the city office for the registration of pregnancy were recruited into the cohort. Their children were followed from birth onwards. Further details of the project have been reported elsewhere.^10^^,^^11^

This study was approved by the ethics review board of the University of Yamanashi’s School of Medicine and was conducted in accordance with the Guidelines Concerning Epidemiological Research (Ministry of Education, Culture, Sports, Science, and Technology and Ministry of Health, Labour, and Welfare, Japan). The Koshu City administrative office collaborated with the authors of this study.

### Measurements

Maternal characteristics and early-life factors were collected from participants’ mothers using a self-reported questionnaire at their pregnancy registration, and interviews were conducted at follow-up by public health nurses in Koshu City.

Participants’ anthropometric data were collected from their birth registration and medical check-up at 3 years of age. Weight z-score ([observed value − mean])/standard deviation [SD]) at birth and at 3 years of age were calculated by gender. Means and SDs were calculated based on all available data from the study sample. Distribution of weights in this dataset was similar to the data of National Growth Survey on Preschool Children in 2000.^12^ Growth in infancy was assessed using the change in weight z-score from birth to 3 years of age. We defined rapid growth in infancy according to the standard proposed by Ong et al,^7^ who indicated that a change in weight z-score exceeding +0.67 between birth and 2 years of age by gender defines rapid growth in infancy. As indicated by Ong et al, z-scores of 0.67 represent the width of each percentile band on standard growth charts (2nd, 10th, 25th, 50th, 75th, 90th, and 98th centile lines); thus, a weight gain exceeding +0.67 SD indicates an increase across at least one of these centile bands.^7^ In our analysis, we used the same cut-off value but a longer age period (birth to 3 years).

### Statistical analysis

We used SAS version 9.3 (SAS Institute, Inc., Cary, NC, USA) for statistical analysis. We used analysis of variance to compare anthropometric indicators at birth and 3 years of age by groups classified according to change in weight z-score. We then examined the crude associations between parental/early-life factors and rapid growth in infancy using logistic regression analysis. The parental factors included maternal age (<25%, <26 years; 25%–75%, 26–32 years; or >75%, >32 years), maternal height (cm; continuous), maternal body mass index (BMI; <18.5 kg/m^2^, 18.5–24.9 kg/m^2^, or ≥25 kg/m^2^), maternal work status (working or not working), maternal smoking habit and alcohol consumption during pregnancy (current smoker/drinker or does not smoke/drink), maternal education level (up to junior high school, high school, or college or higher), and paternal education level (up to junior high school, high school, or college or higher). Participants’ early-life factors included birth weight (<2500 g or ≥2500 g), gestational age (<37 weeks or ≥37 weeks), primiparity (1st birth or other), and breastfeeding during the first 3 and 6 months (exclusive breastfeeding or non-exclusive breastfeeding). The mother provided information on breastfeeding every month from delivery. Exclusive breastfeeding throughout the first 3/6 months was defined as exclusive breastfeeding during the first 3/6 months.

A structural equation model was constructed to examine the pathways between maternal smoking during pregnancy and growth in infancy using AMOS 7.0 (SPSS, Inc., Chicago, IL, USA). The complete-case dataset was used for this analysis. The outcome was growth in infancy (continuous); the independent variables were determined according to the results from the univariate logistic regression analysis above (variables for which *P* ≤ 0.1). Included independent variables were maternal smoking during pregnancy, maternal work status, maternal height and maternal BMI; the mediators were birth weight (g; continuous), gestational age (weeks; continuous), primiparity, and breastfeeding during the first 3 months. Breastfeeding during the first 6 months was not used for analysis because of the large proportion of missing data. The association between the independent variables and the pathways that linked the independent variable to the outcome were determined using an explorative model.

## RESULTS

Maternal information during pregnancy and birth characteristics were collected from 2298 singleton births. Of these, 1926 children undertook the medical checkup at 3 years of age. Therefore, the follow-up rate at 3 years of age was 84%. Two of the 1926 children did not have birth weight data, and 9 did not have weight data at the 3-year checkup. As a result, 1915 children had data available to assess growth speed in infancy. Of these participants, 1524 (66% [1524/2298]; 775 boys and 749 girls) had complete information on maternal and early-life factors. As shown in Table 1, 260 (26.8% [260/1915]) of the boys and 239 (25.3% [239/1915]) of the girls experienced rapid growth in infancy (change in weight z-score >+0.67 SD). These children had lower weight and length at birth than children who did not had rapid growth, in addition to higher weight, height, and BMI at 3 years of age.

**Table 1.** Anthropometric data, classified by change in weight z-score from 0 to 3 years of age

Variables	Change in weight z-score in boys				Change in weight z-score in girls			
	
	<−0.67 SD	−0.67 SD to 0.67 SD	>0.67 SD	*P*-value^a^	<−0.67 SD	−0.67 SD to 0.67 SD	>0.67 SD	*P*-value^a^
Number of participants	288	421	260		259	448	239	
Birth weight (g)	3344 (316)	3032 (290)	2778 (374)	<0.0001	3332 (371)	3002 (309)	2759 (369)	<0.0001
Birth length (cm)	50.1 (1.8)	49.0 (1.7)	48.1 (2.3)	<0.0001	49.7 (2.0)	48.8 (1.7)	47.9 (2.2)	<0.0001
Weight at 3 years of age (kg)	13.5 (1.2)	14.2 (1.2)	15.6 (1.7)	<0.0001	13.2 (1.4)	14.0 (1.3)	15.4 (1.7)	<0.0001
Height at 3 years of age (cm)	94.0 (3.3)	95.3 (3.3)	97.2 (3.7)	<0.0001	93.5 (3.4)	94.7 (3.4)	96.6 (3.4)	<0.0001
BMI at 3 years of age (kg/m^2^)	15.2 (1.0)	15.7 (1.0)	16.5 (1.4)	<0.0001	15.1 (1.1)	15.6 (1.1)	16.5 (1.4)	<0.0001
Weight gain during the first 3 years (kg)	10.1 (1.0)	11.2 (1.0)	12.8 (1.5)	<0.0001	9.9 (1.1)	11.0 (1.1)	12.7 (1.5)	<0.0001
Height gain during the first 3 years (cm)	43.9 (3.1)	46.2 (3.1)	49.2 (3.5)	<0.0001	43.8 (3.2)	45.9 (3.1)	48.7 (3.2)	<0.0001

SD, standard deviation; BMI, body mass index.

^a^Determined using ANOVA.

Variables are presented as mean (SD).

Overall, 774 cases from the original dataset were excluded from the pathway analysis due to lack of information on maternal factors (262 cases), birth characteristics (9 cases), or information during infancy (183 cases). No significant differences in maternal factors (maternal BMI 20.6 kg/m^2^ vs. 20.8 kg/m^2^, *P* = 0.4; maternal height 157.5 cm vs. 157.9 cm, *P* = 0.1; maternal working 45.0% vs. 48.7%, *P* = 0.1; and maternal smoking during pregnancy 7.9% vs. 5.8%, *P* = 0.06) were found between these 774 cases and the 1524 cases included in the pathway analysis.

According to the results of the univariate analysis, low birth weight, preterm birth (gestational age <37 weeks), primiparity, non-exclusive breastfeeding (for the first 3 and 6 months), high maternal height, maternal working, and maternal smoking during pregnancy were significantly associated with rapid growth in infancy (Table 2).

**Table 2.** Maternal and early-life factors related to rapid growth in infancy

Characteristic	*n*	Rapid infancy growth		*P*-value^a^	OR^a^

		Yes	No		
Birth and infancy					
Birth weight, *n* (%)	1915				
<2500 g	122	87 (17.4)	35 (2.5)	<0.0001	8.33 (5.54–12.52)
≥2500 g	1793	412 (82.6)	1381 (97.5)	ref.	ref.
Gestational age, *n* (%)	1909				
<37 weeks	75	60 (12.1)	15 (1.1)	<0.0001	10.78 (7.18–22.73)
≥37 weeks	1834	437 (87.9)	1397 (98.9)	ref.	ref.
Birth order, *n* (%)	1914				
First	772	251 (50.3)	521 (36.8)	<0.0001	1.74 (1.41–2.13)
Second or later	1142	248 (49.7)	894 (63.2)	ref.	ref.
Breastfeeding during the first 3 months, *n* (%)	1743				
Exclusive breastfeeding	698	134 (29.8)	564 (43.6)	<0.0001	0.55 (0.44–0.69)
Other	1045	315 (71.2)	730 (56.4)	ref.	ref.
Breastfeeding during the first 6 months, *n* (%)	973				
Exclusive breastfeeding	308	53 (22.0)	255 (34.8)	0.0002	0.53 (0.38–0.74)
Other	665	188 (78.0)	477 (65.2)	ref.	ref.
Parental					
Maternal age, *n* (%)	1897			0.8	
<26 years (<25th percentile)	369	100 (20.1)	269 (19.2)	0.6	1.04 (0.80–1.36)
26–32 years (25th–75th percentile)	1068	281 (56.5)	787 (56.2)	ref.	ref.
>32 years (>75th percentile)	460	116 (23.3)	344 (24.6)	0.5	0.94 (0.74–1.21)
Maternal height, cm (mean [SD])	1842	158.3 (5.1)	157.7 (5.1)	0.03	1.02 (1.00–1.04)
Maternal BMI, *n* (%)	1689			0.3	
<18.5 kg/m^2^	331	82 (18.7)	249 (19.9)	0.2	0.96 (0.72–1.27)
18.5–24.9 kg/m^2^	1225	314 (71.7)	911 (72.8)	ref.	ref.
≥25 kg/m^2^	133	42 (9.6)	91 (7.3)	0.1	1.34 (0.91–1.97)
Maternal work status, *n* (%)	1898				
Working	917	266 (54.1)	651 (46.3)	0.003	1.37 (1.11–1.68)
Not working	981	226 (45.9)	755 (53.7)	ref.	ref.
Maternal smoking during pregnancy, *n* (%)	1883				
Current smoker	113	44 (8.9)	69 (5.0)	0.002	1.88 (1.27–2.79)
Ex-smoker and non-smoker	1770	448 (91.1)	1322 (95.0)	ref.	ref.
Maternal drinking during pregnancy, *n* (%)	1876				
Current drinker	206	61 (12.4)	145 (10.5)	0.2	1.21 (0.88–1.66)
Ex-drinker and non-drinker	1670	432 (87.6)	1238 (89.5)	ref.	ref.
Maternal education, *n* (%)	1750			0.6	
Up to junior high school	37	10 (2.2)	27 (2.1)	0.9	1.11 (0.53–2.34)
High school	731	198 (43.7)	533 (41.1)	0.8	1.12 (0.90–1.39)
College or higher	982	245 (54.1)	737 (56.8)	ref.	ref.
Paternal education, *n* (%)	1739			0.6	
Up to junior high school	74	21 (4.7)	53 (4.1)	0.6	1.21 (0.71–2.05)
High school	754	200 (44.8)	554 (42.9)	1	1.10 (0.88–1.37)
College or higher	911	225 (50.5)	686 (53.1)	ref.	ref.

OR, odds ratio; SD, standard deviation; BMI, body mass index.

^a^*P*-value and OR were calculated by separate logistic regression for each variable.

The model fit in the pathway analyses was adequate (goodness of fit index 0.99, comparative fit index 0.94, and root mean square error of approximation 0.039). The pathways determined using the exploratory methods, in addition to the standardized regression coefficients and significance, are depicted in Figure. According to the results from the pathway analyses, maternal smoking during pregnancy contributed to lower birth weight. In addition, lower birth weight contributed significantly to rapid growth in infancy. Maternal smoking during pregnancy was also related to infant growth through breastfeeding status during the first 3 months. The indirect standardized effect of maternal smoking through these 3 pathways was 0.04. Besides these pathways, maternal smoking was also directly linked to rapid growth in infancy. The standardized direct effect was 0.06 (*P* = 0.002). Taking all of the pathways into account, the standardized total effect of maternal smoking on infancy growth was 0.11.

**Figure.**
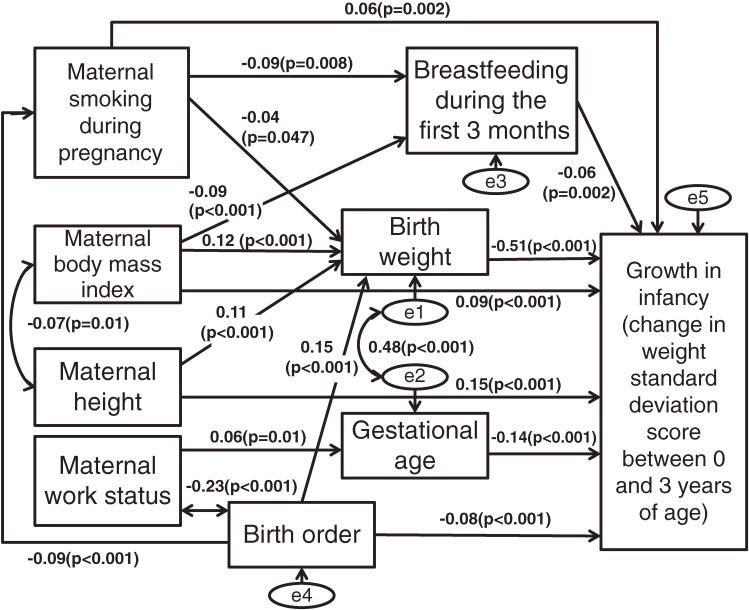
Standardized pathways between maternal smoking during pregnancy and growth in infancy determined using exploratory methods. (goodness of fit index 0.99, comparative fit index 0.94, and root mean square error of approximation 0.039)

## DISCUSSION

In this study, we examined the association between growth in infancy and maternal, birth, and early-life factors. We also explored pathways between maternal smoking during pregnancy and rapid growth in infancy. The results suggested that maternal smoking during pregnancy might be related to accelerated growth in infancy both directly and indirectly through fetal development and early breastfeeding practices. Since previous studies indicated a gender difference in the association between maternal smoking and childhood BMI trajectories,^10^ we also carried out a sub-analysis stratified by gender. However, we did not find a significant gender difference in the association between maternal smoking and infant growth (data not shown).

Previous studies have discussed factors related to rapid growth in infancy. Ong et al reported that children with rapid growth during infancy had lower birth weight and length and were more likely to be the first child than children without rapid growth during infancy. Further, their mothers were more likely to smoke during pregnancy.^7^ Another study by Regnault et al demonstrated that fathers’ heights and BMI as well as their children’s gestational age and feeding methods were related to weight-growth velocity at three months of age.^8^ Further, a study by Nafstad indicated that weight gain during the first year was slower in breastfed children than in those who were not breastfed and faster in children of smoking mothers than those of non-smoking mothers.^6^ Finally, a study by Baird also indicated that infants who were breastfed gained weight more slowly.^13^ These results were similar to those of the present study. However, since these factors were correlated to each other in previous studies, it was difficult to determine the separate contribution of each factor.

Results of pathway analyses showed that lower birth weight and non-exclusive breastfeeding were both mediators of the link between maternal smoking during pregnancy and increased growth in infancy. Primiparity was also related to the pathway. These findings are consistent with previous studies, which indicate that maternal smoking during pregnancy is associated with constrained fetal growth^14^ and low birth weight followed by rapid growth in infancy.^15^ It has been reported that maternal smoking during pregnancy affects fetal development by influencing uterine, umbilical, and fetal cerebral artery blood flow.^16^ Further, catch-up growth occurs in most low-birth-weight infants and is likely to result in childhood obesity.^7^ Birth order may also influence the association between maternal smoking and growth in infancy. Previous studies have confirmed the association between birth order and growth in childhood.^17^^,^^18^ On the other hand, reviews have demonstrated that women who smoke are less likely to breastfeed and breastfeed for a shorter duration than nonsmokers.^19^ Both psychosocial and biological factors are responsible for these associations. Smoking may suppress prolactin secretion and thereby reduce breast milk volume.^20^ Further, formula-fed infants have higher protein intake than breastfed infants, which may stimulate the secretion of insulin. Therefore, these infants may gain weight more rapidly due to the promotion of growth by insulin.^21^^,^^22^

In addition to these indirect pathways through birth weight and breastfeeding status, maternal smoking during pregnancy also contributed directly to infant growth in our model. However, the mechanism for the direct effect was unclear. One possible explanation for this pathway is that maternal smoking during pregnancy results in a fetal biological change that has a relatively small effect on fetal size during pregnancy and a larger effect on growth after birth. Two other analyses using the same Project Koshu data demonstrated that the maternal habit of skipping breakfast during pregnancy did not influence the birth weight of the offspring^23^ but was significantly associated with offspring being overweight and obese at 5 years of age.^11^ Therefore, we speculated that the characteristics of skipping breakfast and smoking during pregnancy may share similar mechanisms. On the other hand, differences in lifestyle and child rearing customs might also explain the differences in infant outcomes between mothers who smoke during pregnancy and those who do not. A study by Rittmueller et al indicated that smokers had higher caloric intake and lower dietary quality than non-smokers.^24^ Another study by Papoz et al showed that infants with mothers who smoke had higher caloric intake than those with non-smoking or formerly smoking mothers.^25^

This study has certain limitations. First, this model may not contain all of the possible pathways between maternal smoking during pregnancy and infant growth. Some factors that were not included in the model might influence the association between maternal smoking during pregnancy and infant growth. Women who smoke during pregnancy may have other biological or personality characteristics that may influence infant growth when compared to nonsmokers, such as socioeconomic status (SES). Low SES is likely to be associated with higher smoking prevalence in mothers^26^ and a higher prevalence of rapid growth in early infancy.^27^ Therefore, low SES might account for the effect of maternal smoking on growth in infancy, at least in part. To clarify this, further studies are needed. Second, when discussing the influence of maternal age on infant growth, we used age categories based on 25th and 75th percentiles. In practice, teenage mothers are more likely to smoke and more likely to give birth to low-birth-weight infants. However, few mothers in this study were teenage mothers. Therefore, it was not possible to clarify the effect of teenage pregnancy in this study. Third, the pathway analyses were conducted using the complete-case dataset, and the proportion of participants with complete data was relatively low (66%). Nevertheless, we compared the characteristics of the participants in the complete dataset to those of the dropped cases. Smoking prevalence was slightly higher among dropped cases than among included cases, but the difference was not significant. Finally, the number of smoking mothers was relatively small because the sample size of this study was not very large and the prevalence of maternal smoking during pregnancy was low. Therefore, findings from this study need further confirmation from larger studies.

Of note, while rapid growth in infancy is strongly associated with childhood obesity, ‘appropriate’ catch-up growth in preterm small-for-gestational-age children has certain benefits.^28^ In this study, however, we were unable to differentiate ‘appropriate’ from ‘inappropriate’ catch-up growth.

In conclusion, maternal smoking during pregnancy may both indirectly, through birth weight and breastfeeding status, and directly influence growth during infancy; however, there may be other implicated pathways that have not been identified yet.

## ONLINE ONLY MATERIAL

Abstract in Japanese.
