# Supplementary material for: Maternal Smoking During Pregnancy and Growth in Infancy: a Covariance Structure Analysis
Source: J Epidemiol. 2015 Jan 5;25(1):44–9. doi: 10.2188/jea.JE20140040 (PMC4275437; doi:10.2188/jea.JE20140040)
Supplement: Abstract in Japanese. [file je-25-044-s001.pdf]

妊娠中の喫煙と出生児の乳幼児期における成長：共分散構造分析による検討

Wei Zheng[1]、鈴木孝太[1]、篠原亮次[2]、佐藤美理[2]、横道洋司[1]、山縣然太朗[1, 2]
山梨大学大学院医学工学総合研究部　社会医学講座[1]

山梨大学大学院医学工学総合研究部附属出生コホート研究センター[2]

背景: 妊娠中の喫煙は胎児の発育制限と出生後の急激な発育に関連していたが、これらの因子間の経路はまだ明らかにされていない。これらの因子における経路の解析は、妊娠中の喫煙が胎内発育、そして乳幼児期の発育に及ぼす影響のメカニズムを解明するために重要である。本研究では、妊娠中の喫煙と出生児における乳児期の成長との間の経路を調べることを目的とした。

方法 : 参加者は、1993 年から 2006 年の間に日本の一地域で生まれた単胎児である。出生体重と 3 歳の体重における標準偏差スコアの変化を主要アウトカムとした。母親の喫煙、他の母親に関する因子（母親の Body Mass Index と就労状況）、出生時の因子（出生体重と在胎週数）と母乳育児のそれぞれが、どのように幼児期の成長と関連しているかを、構造方程式モデリングを用いて検討した。

結果： 1524 人の参加者（男児 775 人、女児 749 人）から欠損値のないデータが得られ、解析モデルは適切と考えられた。出生体重が軽いこと、完全母乳育児でないことは、妊娠中の喫煙と幼児期の急激な発育の関連における媒介因子であった。一方で、母親の喫煙と、乳児期の成長との直接的な関連も観察された（standardized direct effects: 0.06, $p = 0.002$）。すべての経路を考慮した後の、幼児期の成長に与える妊娠中の喫煙の標準化した寄与率は 0.11 であった。

結論 : 妊娠中の喫煙は、出生体重と母乳育児を経由して、間接的に幼児期の成長に影響する経路と、直接的に幼児期の成長に影響する経路の双方が存在する可能性が示された。さらに、他のまだ同定されていない経路が存在するかもしれない。

キーワード：子どもの発達;母親の曝露;喫煙
